# An Analysis of Response Shifts in Teacher Reports Associated with the Use of a Universal School-Based Intervention to Reduce Externalising Behaviour

**DOI:** 10.1007/s11121-019-00999-2

**Published:** 2019-03-07

**Authors:** Aja Louise Murray, Tom Booth, Manuel Eisner, Denis Ribeaud, Karen McKenzie, George Murray

**Affiliations:** 1 Institute of Criminology, University of Cambridge, Sidgwick Avenue, Cambridge, CB3 9DA UK; 2 Department of Psychology, University of Edinburgh, Edinburgh, Scotland; 3 Jacobs Center for Productive Youth Development, University of Zurich, Zürich, Switzerland; 4 Department of Psychology, Northumbria University, Newcastle upon Tyne, England

**Keywords:** Universal interventions, Response shifts, Measurement invariance

## Abstract

School-based psychosocial interventions are a widely used approach to prevent or reduce externalising behaviour. However, evaluating the effects of such interventions is complicated by the fact that the interventions may not only change the target behaviour, but also the way that informants report on that behaviour. For example, teachers may become more aware of bullying behaviour after delivering lessons on the topic, resulting in increased teacher reports of the behaviour. In this study, we used multi-group confirmatory factor analysis to evaluate whether teachers exposed to the Promoting Alternative Thinking Strategies (PATHS) intervention changed the way they reported on child externalising behaviour. Using data from the z-proso study (802 participants; 51% male; 69 teachers), teacher reports of aggressive behaviour, attention deficit hyperactivity disorder and non-aggressive conduct disorder symptoms were compared pre- and post- intervention and across the intervention and control conditions. There was no evidence that teacher reporting was affected by exposure to the intervention. This helps bolster the interpretation of intervention effects as reflecting changes in child behaviour, rather than in the manner of informant reporting.

## Introduction

Externalising behaviours, including aggression, bullying, non-aggressive conduct problems and attention deficit hyperactivity disorder (ADHD) symptoms, create significant challenges for affected individuals, their parents and schools. Externalising problems are the most common reason for referral to mental health services in school children and predict academic, peer, and mental health difficulties, substance abuse and offending later in life (e.g. Buitelaar et al. 2013; Erskine et al. 2016; Ttofi et al. 2011; van Lier et al. 2012). Universal school-based interventions targeted at the whole student population are a widely used approach to preventing or improving child problem behaviour (e.g. Durlak et al. 2011; Ttofi and Farrington 2011). Given that, the majority of the youth population spends up to 6 h a day in school, school-based interventions provide a unique opportunity to engender positive change at, effectively, the population level (e.g. Dunn et al. 2015).

A large body of evidence has investigated the efficacy of school-based interventions for tackling externalising behaviour problems. A meta-analysis by Durlak et al. (2011), for example, identified 112 school-based universal intervention studies targeting conduct problems through social and emotional learning interventions. The pooled effect size of 0.22 indicated a significant but modest improvement in conduct problems. Similarly, in a systematic review and meta-analysis on the topic of bullying, Zych et al. (2015) identified seven meta-analyses evaluating (almost exclusively) school-based interventions for bullying alone. This included one meta-analysis able to analyse 44 programme evaluations (Ttofi & Farrington, 2011), showing an average reduction of around 20% in bullying perpetration and victimisation.

Well-conducted evaluation studies in this area typically involve a comparison of changes in problem behaviour in a treatment versus control group on completion of the intervention. However, there are a number of methodological challenges facing even the most rigorous school-based intervention evaluation studies. These include widely recognised difficulties such as recruiting schools willing to be in the control group, diffusion of effects into control units, evaluator biases, attrition and identifying the ‘active ingredients’ when interventions combine multiple components (e.g. Chalamandaris and Piette 2015). For many issues, study design features are possible that at least in principle solve the most serious threats to the validity of a trial. For example, to prevent contamination of control participants by the intervention, randomisation can be done at the level of the school; to encourage control-group participation, schools may be randomised to a wait list to receive the intervention later. Similarly, evaluations by researchers independent of the developers of a programme can help overcome evaluator biases, while meta-regressions in meta-analytic studies can help solve the problem of identifying the most effective components so long as there is some variation in component combinations across programmes.

One methodological issue where there remains a need for further research, however, relates to a phenomenon termed ‘response shifts’ (e.g. Oort et al. 2009). Response shifts can be defined as changes in the measurement of a construct of interest, e.g. ‘aggression’, that are attributable to changes in the conceptualisation, awareness, frame of reference or understanding of that construct. Crucially, these changes affect responses to questionnaire measures and can be mistaken for or mask changes due to interventions. For example, it has been proposed that some school-based interventions fail to show positive effects because they sensitise informants to the behaviours that the intervention seeks to change. This, it is argued, can result in increased reporting of those behaviours post-intervention (for a discussion, see Chalamandaris and Piette 2015).

Response shifts can occur merely as a result of repeated administration of a questionnaire (analogous to practice effects on cognitive tests; e.g. Lievens et al. 2007); however, they may be particularly liable to occur in the context of psychosocial interventions. Recipients of psychosocial interventions are likely to acquire new knowledge on and/or may be encouraged to think differently about a target construct (Murray et al. 2018). For example, improved awareness may make recipients better able to detect behaviours or symptoms, to discriminate between different behaviours (e.g. reactive vs proactive aggression), or to better understand what a questionnaire item is asking. The participants may also engender more fundamental re-conceptualisations of the constructs being assessed (e.g. if an informant no longer conceptualises ‘stealing’ as a marker of aggression after being exposed to educational materials). When such changes occur, responses to questionnaire items measuring the construct may change even if the target behaviour has not.

Most evidence of response shifts has come from quality of life and mental health research (e.g. Fokkema et al. 2013; Murray et al. 2018). For example, Fokkema et al. (2013) evaluated response shifts in the Beck Depression Inventory following a psychotherapeutic intervention. They found evidence to suggest that, for the same underlying severity of depression, after therapy, some items were scored higher. In addition, item residual variances were overall smaller after therapy. The authors interpreted these findings as suggesting that therapy made people more aware of their symptoms and better able to report on them. They also highlighted that these measurement differences would have resulted in biased estimates of the treatment effect had they not been identified and steps taken to ensure the measurement changes were appropriately modelled.

Despite their potential to confound the estimation of treatment effects in school-based interventions, there has been an almost complete lack of research into response shifts in this area. In this study, we, therefore, evaluate response shifts in the context of a widely used school-based intervention programme. The study represents a re-analysis of a previously reported intervention evaluation examining the Promoting Alternative Thinking Styles (PATHS) programme within the Zurich Project on Social Development of Youth study (z-proso; Malti et al. 2011). PATHS is a social and emotional learning-based intervention administered by teachers. It involves lessons on problem-solving, social relationships, self-regulation, rule understanding, emotion understanding and positive self-esteem. As administered in z-proso, it targeted three subdimensions of externalising behaviour: ADHD symptoms, aggressive behaviour and non-aggressive conduct disorder symptoms. In z-proso, Malti et al. (2011) found some significant improvements on externalising behaviour in PATHS relative to the control group (*d* = − 0.42 for aggression; − 0.46 for ADHD) based on teacher reports but no significant improvement on non-aggressive conduct problems. However, as argued above, observed changes pre- versus post-intervention (or lack thereof) may not entirely reflect treatment effects if teachers exposed to the intervention material changed the way they perceived and/or reported on child behaviour as a result. For example, the programme may have increased their own understanding of certain child behaviours, making them less likely to attribute the motive as aggressive. Evaluating whether such changes in teacher reports occurred was the focus of the current study.

## Method

### Participants

Participants were in the first and/or third waves of z-proso, an ongoing longitudinal cohort study of crime and aggression based in Zurich. The study began in 2004 when participants were entering the first grade. All children entering the first grade (aged ~ 7) in 56 selected schools were invited to participate via their parents who were offered an incentive worth 30USD. Schools were selected based on a stratified random sampling procedure that took into account school size and location. This gave a total target sample size of *n* = 1675. Over the course of the study, 1572 children (currently spanning ages 7 to 17) have provided data at some point. Full details of recruitment, assessment, attrition and other information on the study can be found in previous publications (e.g. Eisner and Ribeaud 2007; Eisner et al. 2019) and at the study website: http://www.jacobscenter.uzh.ch/en/research/zproso/aboutus.html. Previous analyses have suggested that the sample can be considered approximately representative of the underlying same-aged Zurich population (e.g. Eisner et al. 2019). The main exception is that children whose parents do not speak German as a first language were slightly more likely to decline to participate.

Waves 1 and 3 of z-proso were selected for the present study because they fell at the baseline and immediate follow-up of the PATHS intervention (described below). The sample utilised comprised 802 participants (51% male) and 69 teachers. This represents a subsample of the full wave 1 and 3 samples because some participants (39%) were rated by teachers who received providing baseline data after the date of PATHS training. These individuals were excluded from the present analyses because baseline scores in the treatment group may already have been affected by teachers’ exposure to the intervention. To maintain comparability between control and treatment groups, controls whose baseline measurements were obtained prior to the date of PATHS training were also excluded. The participants were divided approximately evenly between the PATHS intervention (*n* = 416) and control (*n* = 386) group.

### Intervention

The z-proso study included two universal preventive interventions: PATHS and Triple-P. Participants were assigned to one of four groups overall: the group that received no intervention (control), the group that received only the PATHS intervention (PATHS), the group that received only the Triple-P intervention (Triple-P) and the group that received both the PATHS and Triple-P intervention (PATHS+Triple-P). Assignment to groups was at the school level, with a randomised block design (14 blocks of 4 schools) to help ensure balance. Schools were selected as the randomisation unit to minimise diffusion effects and because PATHS is presumed to work best this way.

In this study, we compared teacher ratings on children who received the PATHS intervention (PATHS and PATHS+Triple-P groups) with those who did not (Triple-P and control groups). The purpose was to determine whether exposure to the PATHS intervention affected the way that teachers rated student behaviours in a manner consistent with response shifts. Those exposed to the PATHS intervention are henceforth referred to as the ‘treatment’ group and those not exposed are referred to as the ‘control’ group. Response shifts related to Triple-P exposure were not analysed in the current study because the informants (the teachers) did not deliver this intervention, thus we had no reason to believe that this intervention should have affected teacher reports of externalising.

PATHS is an evidence-based intervention which aims to reduce externalising behaviour in primary school-aged children through improving social competence (Greenberg & Kusche, 2002). It has been evaluated in a number of trials where it has generally shown positive but modest effects (e.g. Crean & Johnson, 2013; Riggs et al. 2006). Evaluations of PATHS within z-proso have been reported in Malti et al. (2011) and Averdijk et al. (2016). Malti et al. (2011) reported significant effects of the PATHS intervention on aggressive behaviour (*d* = − 0.42) and ADHD (*d* = − 0.46) when followed up at wave 4 (grade 4) based on the teacher reports. However, there were no significant effects based on self-reports and only a very small effect based on parent reports limited to aggressive behaviour. Averdijk et al. (2016) examined the long-term effects of PATHS. They found little evidence for protective effects of PATHS by age 13–15; only 1 of 13 outcomes related to externalising behaviour (reduced police contacts) was associated with the intervention at this stage, with an effect size of − 0.22.

**Fig. 1:**
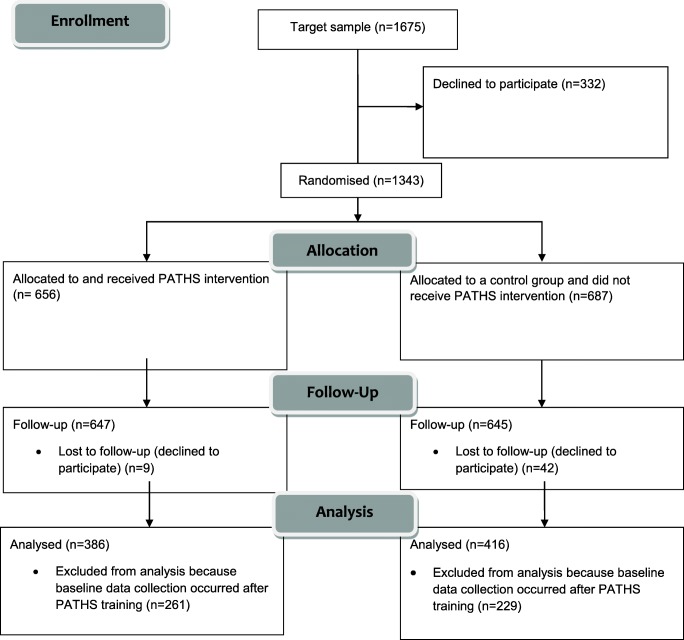
Intervention flow diagram

**Fig. 2:**
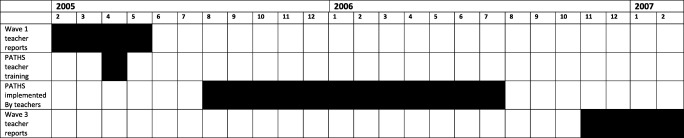
Project timeline

A flow diagram summarising numbers of participants recruited, allocated, measured at baseline and follow-up and included in analyses is provided in Fig. 1. These numbers refer to the participants analysed as part of the present study. A flow diagram for the main intervention evaluation can be found in Averdijk et al. (2016). The timeline of the project up to wave 3 is shown in Fig. 2. PATHS was administered between waves 2 and 3 of the z-proso study in years 2005–2006, corresponding to school grades 2 and 3. During this time, it was made a compulsory part of the curriculum for the schools assigned to the intervention condition. It represented a 1-year programme of 46 primary lessons and a number of additional secondary lessons. The classes were around 67 min per week across an average of 2.4 sessions. The version used was similar to the one used in the Fast Track Project (Bierman 1996) but with adaptations to the Swiss school system. The materials were extensively tested in a pilot study, comprehensive details of which can be found in Eisner et al. (2006). The PATHS course deals with problem solving skills, social relationships, self-regulation, rule understanding, emotion understanding and positive self-esteem.

PATHS was administered by the normal class teachers who also provided ratings on the children. The teachers received a 2-day training course administered by five trainers and also attended a refresher seminar mid-term. The initial training course took place in April 2005. The trainers themselves were trained and supervised by a high-level PATHS expert. Trainers visited the classrooms and discussed the lessons with the teachers between four and six times and during the intervention. Teacher reports suggested that the intervention was generally received well by them. The majority (88%) evaluated the curriculum positively, with 85% rating the training as good or very good and 61% rating the training as supportive.

Implementation of the intervention was monitored and assessed using teacher and child questionnaires and trainer observations. Reports by the trainers suggested that an average of 27/30 mandatory lessons were completed and implementation quality was rated as high. Scores on quality of classroom leadership, child motivation and teaching of PATHS averaged 88%, 82% and 74% respectively.

### Measures

Aggressive behaviour, ADHD and non-aggressive conduct disorder symptoms were measured using the Social Behavior Questionnaire (SBQ; Tremblay et al. 1991). Administration was in the German language, reflecting the official language of the region in which the study took place (English translations are provided in Table 1). Responses were provided on a 5-point Likert scale from (translates to) ‘never’ to ‘very often’. The SBQ was administered to teachers in paper and pencil format pre- and post-intervention. Previous psychometric evaluations of the SBQ items used in the current study have supported their reliability and validity (e.g. Murray et al. 2017a, b, Murray et al. 2018).

**Table 1:** Item contents for the Social Behavior Questionnaire (SBQ)

Item number	Item content
Attention deficit hyperactivity disorder (ADHD)	
10	is impulsive, acts without thinking.
11	has difficulty awaiting turn in games or groups.
12	cannot sit still, is restless, or hyperactive.
13	fidgets.
14	cannot settle to anything for more than a few moments.
15	is distractible, has trouble sticking to any activity.
16	cannot concentrate, cannot pay attention for long.
17	is inattentive.
18	gives up easily.
Non-aggressive conduct disorder symptoms	
24	steals at home.
25	steals outside the home.
26	destroys his\her own things.
31	destroys things belonging to his\her family, or other children.
32	tells lies and cheats.
Aggressive behaviour	
19	When mad at someone, tries to get others to dislike that person.
20	When mad at someone, becomes friends with another as revenge.
21	When mad at someone, says bad things behind the other’s back.
22	When mad at someone, says to others: let us not be with him\her.
23	When mad at someone, tells the other one’s secrets to a third person.
23	gets into fights.
34	physically attacks people.
35	kicks, bites, hits other children.
36	is cruel, bullies, or is mean to others.
37	threatens people.
39	kicks, bites, hits his\her mother.
50	encourages other children to pick on a particular child.
51	tries to dominate other children.
52	scares other children to get what he\she wanted.
53	reacts in an aggressive manner when teased.
54	reacts in an aggressive manner when something was taken.
55	reacts in an aggressive manner when contradicted.

### Statistical Procedure

Response shifts can be evaluated using a multi-group longitudinal confirmatory factor analysis measurement invariance approach. Measurement invariance is when the conditional distribution of observed scores is independent of a ‘violator’ of interest, such as sex or measurement wave. Here, we are concerned with two potential violators: time (pre- vs post-intervention) and group (PATHS vs control). In practice, full measurement invariance is difficult to test but the closely related property of factorial invariance can be easily evaluated within a confirmatory factor analysis framework. Response shifts would be detected as violations of factorial invariance over time in the treatment-exposed group. For comparison, violations of factorial invariance over time in the control group can be tested to ensure that changes are due to intervention exposure, rather than any other changes that occurred over the treatment period and/or to the re-administration of the questionnaire.

**Fig. 3:**
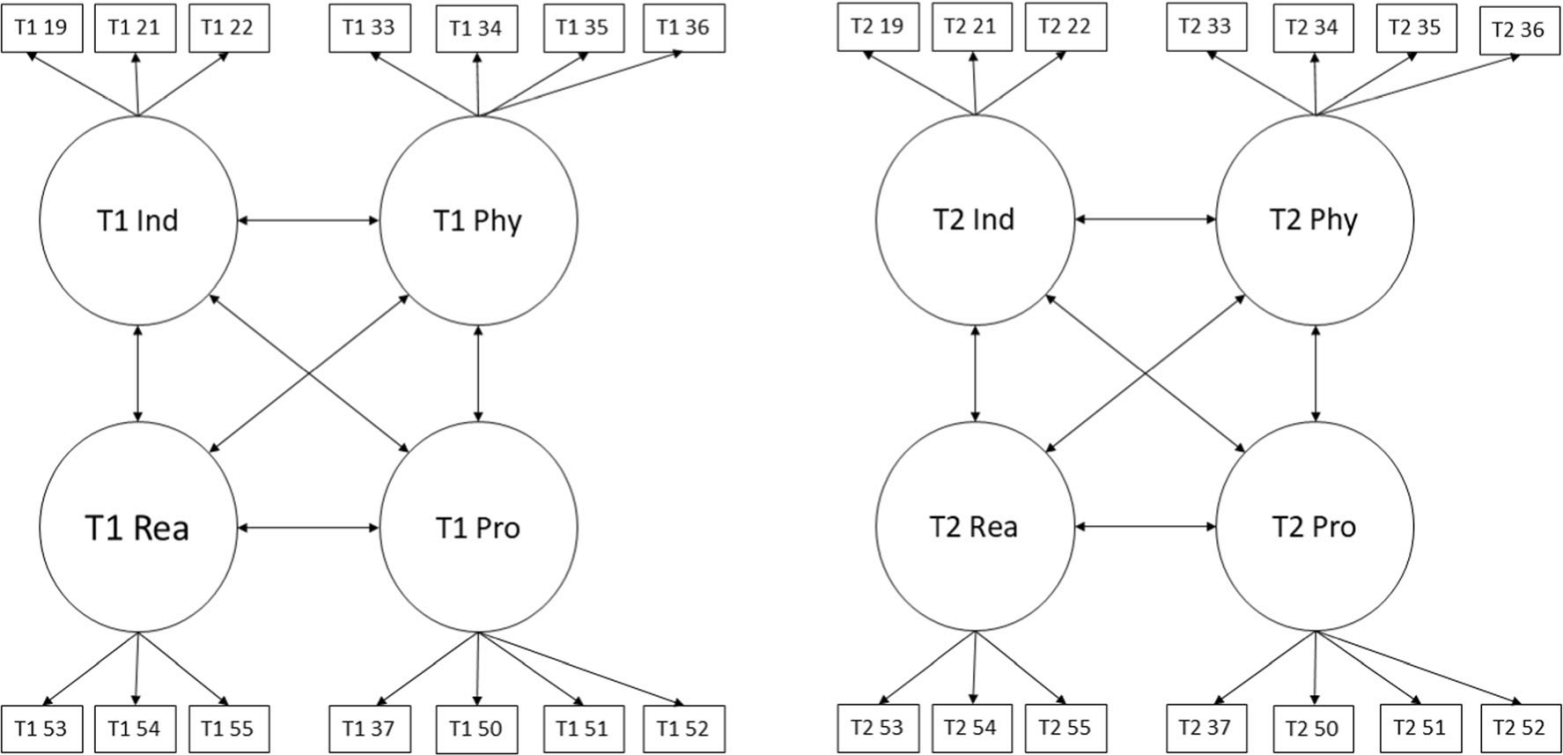
Configural model for aggressive behaviour. Ind, indirect aggression; Phy, physical aggression; Rea, reactive aggression; Pro, proactive aggression; T1/T2 indicate time point 1 (baseline) versus time point 2 (follow-up). Item numbers for the SBQ are also indicated in the rectangles representing the items. Error variances and covariances and mean structure have been omitted for clarity

**Fig. 4:**
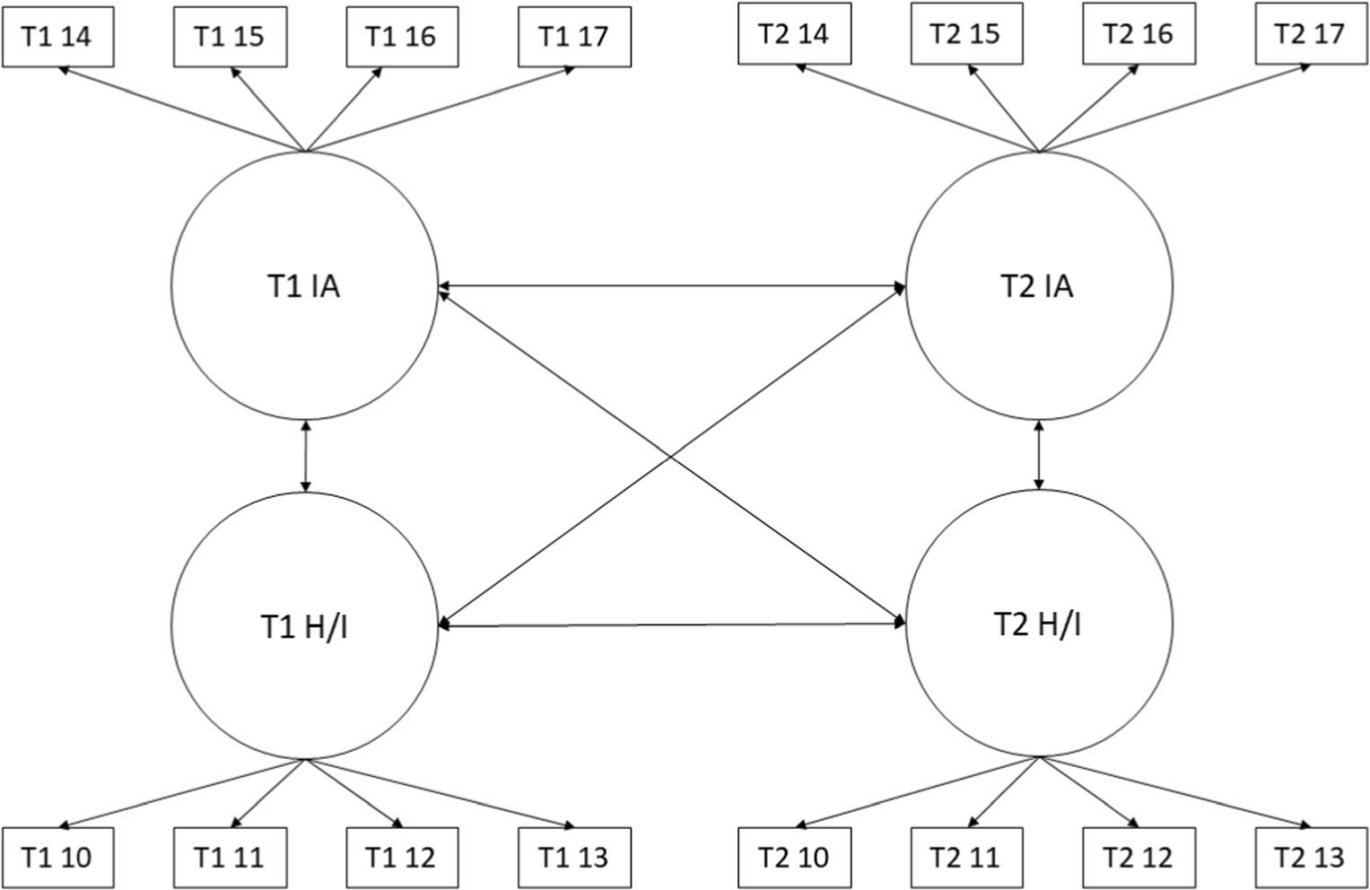
Configural model for attention deficit hyperactivity disorder (ADHD). IA, inattention; H/I, hyperactivity impulsivity; T1/T2 indicate time point 1 (baseline) versus time point 2 (follow-up). Item numbers for the SBQ are also indicated in the rectangles representing the items. Error variances and covariances and mean structure have been omitted for clarity

**Fig. 5:**
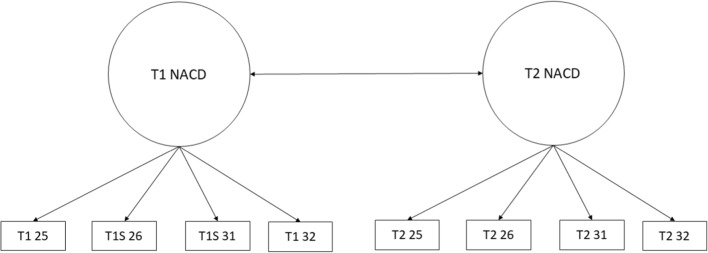
Configural model for non-aggressive conduct disorder symptoms. NACD, non-aggressive conduct disorder, T1/T2 indicate time point 1 (baseline) versus time point 2 (follow-up). Item numbers for the SBQ are also indicated in the rectangles representing the items. Error variances and covariances and mean structure have been omitted for clarity

We began by fitting a configural model for each set of behavioural dimensions separately (aggressive behaviour, ADHD and non-aggressive conduct disorder symptoms). Configural models for each behavioural dimension are summarised in Figs. 3, 4, and 5. Aggressive behaviour was defined by four correlated latent factors: physical aggression, indirect aggression, reactive aggression and proactive aggression. ADHD was defined by two correlated factors: inattention and hyperactivity/impulsivity. Non-aggressive conduct disorder symptoms were defined by a single latent variable. These configural models were multi-group models with the relevant behavioural dimension at waves 1 and 3 represented by latent factors. To identify and scale latent factors, the mean and variance of wave 1 control group latent factors were fixed to 0 and 1 respectively. In addition, one loading and intercept for each factor at wave 1 was fixed equal to the corresponding loading and intercept at wave 3 for the control and in both waves in the treatment group. Finally, covariances between latent factors and residual covariances between the same items measured at waves 1 and 3 were freely estimated.

We used the criteria offered by Chen (2007) to judge whether measurement invariance held at the configural, metric, scalar and residual level. Specifically, configural invariance was judged to hold if the configural model showed acceptable fit according to conventional fit criteria. Metric invariance was judged to hold if the addition of equality constraints in loadings resulted in a decrease of no more than 0.010 in the comparative fit index (CFI), an increase of no more than 0.015 in the root mean square error of approximation (RMSEA) and an increase of no more than 0.030 in the standardised root mean square residual (SRMR). Scalar invariance was judged to hold if the addition of equality constraints on intercepts resulted in a decrease in CFI of no more than 0.010, an increase in RMSEA of no more than 0.015 and an increase in SRMR of no more than 0.010. Strict invariance was judged to hold when CFI decreased by less than 0.010, when RMSEA increased by less than 0.015 and SRMR increased by less than 0.010 with the addition of strict invariance constraints. If invariance did not hold at any stage, we used modification indices to guide the identification of untenable constraints and remove them to achieve a partial invariance model. All models were fit in Mplus 7.13 using robust maximum likelihood estimation to take account of the clustering within teachers (Muthén and Muthén 2013). Mplus scripts can be obtained by emailing the first author.

## Results

### Aggressive Behaviour

The configural model for aggressive behaviour showed acceptable fit (CFI = 0.908; RMSEA = 0.074; SRMR = 0.074). Adding metric invariance constraints resulted in only a slight change in model fit (CFI = 0.908; RMSEA = 0.072; SRMR = 0.081) which was consistent with metric invariance holding. With the addition of scalar invariance constraints, model fit again changed only slightly (CFI = 0.901; RMSEA = 0.073; SRMR = 0.081), suggesting that scalar invariance held. Adding strict invariance constraints resulted again in only a slight change in fit (CFI = 0.902; RMSEA = 0.071; SRMR = 0.083), suggesting that strict invariance held.

### ADHD

The initial configural model for ADHD showed estimation problems that could only be resolved by removal of the clustering within teachers. The configural model with no clustering showed reasonable fit (CFI = 0.949; RMSEA = 0.077; SRMR = 0.056). Adding metric invariance constraints resulted in no change in CFI, a slight improvement in RMSEA and a slight deterioration in SRMR (CFI = 0.949; RMSEA = 0.073; SRMR = 0.058), suggesting that metric invariance held. Adding scalar invariance constraints to the model, again, little affected fit (CFI = 0.947; RMSEA = 0.074; SRMR = 0.058), suggesting that scalar invariance held. Adding strict invariance constraints did not suggest any violations of strict invariance (CFI = 0.946; RMSEA = 0.076; SRMR = 0.058).

### Non-aggressive Conduct Disorder Symptoms

The configural model for non-aggressive conduct problems fit well (CFI = 0.951; RMSEA = 0.059; SRMR = 0.066). Adding metric invariance constraints resulted in a change in fit that did not indicate violation of metric invariance (CFI = 0.957; RMSEA = 0.048; SRMR = 0.089). Adding scalar invariance constraints reduced fit again (CFI = 0.951; RMSEA = 0.047; SRMR = 0.090), but not enough to suggest violations of scalar invariance. Finally, adding strict invariance constraints suggested violation according to SRMR but not CFI or RMSEA (CFI = 0.972; RMSEA = 0.031; SRMR = 0.102). We thus inspected modification indices and expected parameter changes to identify sources of misfit; however, all were small. Given this, the fact that the change in SRMR only slightly exceeded the pre-specified threshold and the fact that fit actually improved with the addition of strict invariance constraints for RMSEA and CFI, we concluded, on balance, that strict invariance held for non-aggressive conduct problems.

## Discussion

In this study, we evaluated whether teachers exposed to a universal preventive intervention programme altered the manner in which they reported on child behaviour. Response shifts of this type have the potential to mask or inflate intervention effects if they are not appropriately modelled. Previous studies have identified response shifts due to psychosocial interventions but this possibility has not yet been addressed in school-based interventions targeting externalising behaviour. We found no evidence for response shifts, thus bolstering the original interpretation of the intervention effects previously reported and supporting the use of the SBQ in intervention studies.

Although methodological issues have received a lot of attention in school-based intervention research, the specific question of whether and how response shifts could confound estimates of treatment effects has thus far been under -researched. This is in contrast to quality of life and related research areas where considerable research has been focussed on identifying and understanding the methodological and theoretical implications of response shifts (e.g. Nolte et al. 2016). Issues of response shifts are, however, also applicable to school-based interventions where the informant on behaviour change has some exposure to the intervention. In the case of the current study, teachers were involved both in administering the intervention and in rating the behaviour of the child before and after the intervention. They could thus be considered vulnerable to the effects of response shifts.

There was, however, no evidence of response shifts over time for any of the dimensions measured, either in the control group or in the intervention group. In fact, strict invariance was achieved for all of aggression, non-aggressive conduct problems and ADHD across both time and treatment groups. This result thus supports the use of the SBQ as a measure of externalising in school-based interventions. Resistance to response shifts is a potentially important but rarely discussed criterion by which measures in intervention studies could be chosen. By demonstrating this property in the SBQ, our study adds to the existing evidence base for the SBQ as a measure of choice for measuring externalising problems across a wide range of ages and across teacher-, parent- and self-report formats (e.g. Tremblay et al. 1991; Murray et al. 2017a, b; Murray et al. 2018).

The fact that metric invariance was achieved over time and across the intervention and control groups is consistent with the idea that the concepts of interest have retained the same meaning after the intervention. If fundamental shifts in the loadings had been observed in the treatment group over time, an argument could have been made that the same construct was not being measured at the two time points. Similarly, the fact that scalar invariance was achieved suggests that for the same underlying levels of externalising behaviour, teachers endorsed the same response option before and after the intervention. This helps to rule out the possibility of sensitisation effects whereby, even if behaviour does not change, a respondent becomes more likely to rate the behaviour as present or more severe because the intervention engenders awareness. Finally, the fact that strict invariance held suggests that teachers were no more or less reliable raters of child behaviour after the intervention or across the treatment and control groups.

The lack of response shifts is also relevant for concerns about the effects of embedding interventions in observational studies. Trials within longitudinal observational studies have a number of advantages, including, for example the ability to examine long-term changes due to the intervention (e.g. Averdijk et al. 2016). Concerns are, however, sometimes voiced that interventions can ‘contaminate’ observational studies by, for example changing the natural levels of relationships between or understanding of study variables among those exposed to the intervention. Our results suggest that, at least for teacher reported externalising constructs (aggression, ADHD and non-aggressive conduct problems), this is not a major issue. Further work would be required to determine whether the intervention affected the structural relations between constructs.

The lack of response shifts in the current study is encouraging for the measurement of change within school-based interventions. However, all evaluation studies should ideally check rather measurement invariance over time when estimating treatment effects. Failing to detect them when present could result in misleading treatment effects, while checking for measurement invariance strengthens the interpretation of intervention effects (or lack thereof) as genuine effects of the intervention. In fact, when violations of measurement invariance are detected, they can often be dealt with relatively straightforwardly. Provided there are a sufficient number of items per construct that show invariance, a lack of invariance can often be resolved by using a partial invariance model. Further, when violations of invariance do arise, they may reveal substantively interesting effects. For example, analyses of invariance across cultures have identified unanticipated differences in the way that items are understood (Nye et al. 2008). In intervention contexts, they may provide further insights into how the intervention affects awareness, knowledge or conceptualisation of the behaviours that the intervention seeks to change.

## Conclusions

Informant response shifts are a potential source of bias in psychosocial school-based interventions. However, in the z-proso study, there was no evidence for response shifts in teacher reports of aggression, non-aggressive conduct problems and ADHD associated with the PATHS intervention.
